# More Life and Fuller

**Published:** 1890-09-06

**Authors:** 


### The Doctor's Pulpit.

### MORE LIFE AND FULLER.

Very imaginative poets, and young lady novelists who give their blushing love to Greek gods, are constantly telling us that we ought to be taller, more muscular, more active in running and leaping and fighting, more faithful in love and more dauntless in war than seems to be possible for the young men of this generation. It must be confessed that the grand heroes of the school-girl's vivid fancy are uncommonly difficult to find, out of books. Although this is a cynical age, all men are not cynics. Indeed very few Englishmen are cynics at heart. It requires a certain level of intellectual sterility and a considerable degree of thinness of blood for a man to be moderately successful in the cynical line of business. The typical Englishman is by no means excessively intellectual; but neither is he thin blooded. Almost every honest John Bull can admire a hero when he sees him. But then the hero must be particularly real and substantial in order to make appeal to his somewhat stolid mind. Shaw, the Life Guardsman, who killed a dozen Frenchmen with his sword at Waterloo, and Grace, the cricketer, would admirably suit the prevailing English ideas of the heroic if their wits were equal to their muscles. There is real sense in this. Every man who can think freely, without being influenced by his own personal circumstances, sees quite clearly that there is an advantage in size, strength, and activity. If the big man could have big wits, and a big heart, he would not only please young ladies, but boys, and even men of his own age. The Greek god idea is not at all bad; only an English god would be much better. The type of man that has resulted from the interbreeding of the Anglo-Saxon and the Norman is a much finer all-round specimen of humanity than was the best of the Greeks. He is an honester man, and a prouder; prouder in the worthiest sense. His intellect is perhaps a little less agile, but it is not weaker, it is stronger; and it is steadier and much more resolute. As the noblest horse is to the boldest lion, so is the modern Englishman to the ancient Greek.

In the present state of things a physical basis is the indispensable condition of all kinds of excellence. The wit cannot be witty without his body, neither can the saint be saintly. Since man must have a body it is best that he should have one that is strong and of noble form. There is no merit in weakness, and no advantage; and ugliness cannot commend itself to any wholesome judgment. With the demand of the ardent poet and novelist for "more life and fuller" we entirely agree; only we shall ask to be permitted to choose for ourselves the quality of the life which is to be bestowed upon us in largely increased measure.

One of the most obvious generalisations which scientific thinking can formulate is, that all creatures are excellent and happy according as they live after their kind. A lion, for example, would be stunted in growth, diminished in sprightliness and activity, and degenerate in form, if he were compelled to live under the conditions which so admirably suit the domestic cow. The lion is best when he is in the enjoyment of complete liberty, and when the conditions of his life are such as to demand his keenest intelligence and the exercise of his greatest physical activity in the acquisition of food. Under such circumstances he is in most cases a noble lion nobly formed. It is almost more essential for a man to live after his kind than for a lion. In the proportion in which a man is by his nature higher than the lion, in that proportion is he capable of more varied and deeper degradation. From the standpoint of outward form an ugly, misshapen, and vicious dwarf is to other men an object of much greater aversion than a small and stunted wild beast. The very name "man" connotes the ideas of thought, beauty, and majesty.

Speaking generally, physical beauty, health, and strength ought to be exceedingly desired by men and women. People should put as much purpose, intelligence, and effort into the search for physical vigour and nobility of form as they put into their professional work. "The life is more than meat and the body than raiment." This was said by an entirely unconventional thinker, who saw much deeper than the average metaphysician and mechanical gerund grinder of his time. The critics of his day probably did not think much of him because he did not reason according to their rules. We all know now, even our critics, how very foolish and inconsequent those ancient reasoners were. But modern critics are much the same as ancient; with them, also, whatever is not according to rule is an ugly duckling, and to be driven away. The average modern man does not live in the exercise of a free and unconventional intelligence. His is not a royal mind which thinks and chooses among the things of life. He does what he feels he must, instead of what he should. Carlyle says, that, "Though we are compassed about by necessity, yet is the meaning of life itself but freedom, voluntary force." The medical mind desires this large and royal freedom for men, and desires it in order that every man may be able to choose for himself that way of life which will give him the greatest beauty of form, the fullest measure of physical vigour, the firmest resolution for duty, and the largest amount of rational pleasure in the exercise of all his functions.

But that is only a part of what is to be desired for men. If modern science has made one fact clearer than another about man it is this: that the differentiating characteristic which most decisively marks him off from all other mammals is his mind, his mental and moral endowment. The distinctive feature of the lion is that he is a splendid carnivore; of the horse, that he is a beautiful living velocipede. The one unavoidable mark in man that strikes a scientific classificator of mammals is not beauty of form, strength of limb, nor swiftness of foot, but mind; thinking, ruling, majestic mind. If man is to have "more life and fuller," it must first and chiefly be mental life. Even a high development of the moral life may fail to present us with a man of the highest order, unless the morality be governed, informed and expanded to its broadest measure by intellect. Mind is the indispensable basis of morals. Without mind there is no such thing as morality. Who thinks of speaking of a moral pig, or a moral crocodile? To exalt the physical at the expense of the mental is to degrade the man; to put the moral before the mental, or in place of it, is to dwarf, and in that sense also to degrade the man. What man or woman ever lived who did not daily sigh over his own mental feebleness and inconsequence? Carlyle gave expression to one of the most pathetic facts of experience when he said: "Ever, to be weak is the true misery." But even that misery may be so tempered by a broad and forward-looking intelligence that it shall cease to be a misery.

The large and strong mind has in it the possibility of the fullest and completest life. It chooses among physical instincts, pleasures, and duties; it rules among them. Without such choice and rule there is little probability of high and permanent physical health, and no hope at all of mental repose. The capable mind chooses, too, among the different lines of life that open out before a man; selects duties for special devotion; resolves both what it can do and what it ought to do. Even in the region of religion the sight and control of the mind are quite as indispensable as in the department of the animal instincts. Millions of persons of the best intentions fail of living successfully for themselves and beneficially for other men for no other reason than want of clearness and strength of mind. "More life and fuller" is the natural cry of all strenuous souls; we can only have life larger and fuller by seeking that sort of expansion and concentration which nature has ordained for creatures of our kind. The powerful brain, the resistless will, the brotherly purpose towards all men, will give us the most and the fullest life which any of us can stand under.

